# Investigation of the properties of polyester blended knitted fabric dyed with luminescent dyes as a highly visible fabric

**DOI:** 10.1038/s41598-025-97356-x

**Published:** 2025-04-27

**Authors:** Marwa A. Ali, Khaled M. Seddik, Ahmed G. Hassabo, Nancy S. El-Hawary

**Affiliations:** 1 Spinning and Weaving Engineering Department, National Research Centre (Scopus affiliation ID 60014618), Textile Research and Technology Institute, 33 El-Behouth St. (former El-Tahrir str.), Dokki, P.O. 12622, Giza, Egypt; 2 Clothing and Knitting Industrial Research Department, National Research Centre (Scopus affiliation ID 60014618), Textile Research and Technology Institute, 33 El-Behouth St. (former El-Tahrir str.), Dokki, P.O. 12622, Giza, Egypt; 3 Pre-treatment, and Finishing of Cellulose-based Fibres Department, National Research Centre (Scopus affiliation ID 60014618), Textile Research and Technology Institute, 33 El-Behouth St. (former El-Tahrir str.), Dokki, P.O. 12622, Giza, Egypt; 4 Dyeing, Printing and Intermediate Auxiliaries Department, National Research Centre (Scopus affiliation ID 60014618), Textile Research and Technology Institute, 33 El-Behouth St. (former El- Tahrir str.), Dokki, P.O. 12622, Giza, Egypt

**Keywords:** High visible fabric (HVF), Luminescent dye, Fluorescent effect, Blended polyester, Knitting technique, Chemistry, Materials science

## Abstract

Recently, many challenges have emerged in the dyes and textile fields to keep pace with the needs of the modern era while achieving safety and comfort during usage. Where the fluorescent dye caused a major boom in the field of dyes and was used to paint many surfaces for exciting and to attract attention purpose. They were utilized on textiles, particularly those composed of synthetic materials employed in hazardous areas such as traffic roads, where they become more visible upon exposure to light. Therefore, this research aims to investigate the color fastness, physio-mechanical properties, and UV resistance of fabrics produced by knitting techniques from polyester blended with cotton or bamboo and dyed with fluorescence dyes as dispersing yellow and red dyes. The evaluation results of dyed fabrics pointed to the dispersed red dyes improved the physio-mechanical, comfortable and colorfastness properties of polyester/cotton samples compared to polyester/bamboo samples except bursting resistance, while the dispersed yellow dyes considerably enrich the ultraviolet protections of polyester/bamboo samples compared to polyester/cotton.

## Introduction

High-visibility clothing is highly luminescent in its natural matt state and stands out against any background; it is also known as hi-vis or hi-viz. It is most commonly worn on the torso and arms of a peace garment. High-visibility clothing was used as personal protective equipment. As a result, it is subject to health and safety guidelines. Disperse Yellow and orange are the most common colors used in high-visibility vests to ensure adequate luminescence compliance with standards such as ISO 20,471^1,2^.

Hi-vis clothing is a type of personal protective equipment designed to prevent accidents caused by people who are not seen. As a result, it is typically worn in occupations that create hazardous situations when working in low visibility, high-risk environments. Railway and road workers, airport workers, and emergency service personnel are examples of occupations that require hi-visibility clothing. Cyclists and motorcyclists may use high-visibility clothing to improve visibility when riding in traffic, racing, or driving on highways. Furthermore, in some areas, hunters may be required to wear hi-vis clothing to avoid accidental shooting^3,4^.

Fluorescent dyes differ from regular dyes in that they can absorb light at one wavelength before emitting light at a longer wavelength, resulting in a visible glow or fluorescence. This property makes fluorescent dyes useful for a variety of applications, including biological imaging, materials science, and security features. Regular dyes do not exhibit fluorescence and are used to color materials that do not emit light. Furthermore, a regular dye can absorb visible light (also known as “white” light, which is a combination of all wavelengths detectable by the human eye) and then reflect some of it in the same part of the spectrum. A fluorescent dye can absorb non-visible wavelengths (usually ultraviolet) and then reflect them back into the visible spectrum. This can make fluorescent dyes appear brighter than they actually are^5^.

The three main types of luminescence are fluorescence, phosphorescence, and chemiluminescence. Fluorescence and phosphorescence are two types of photoluminescence. In photoluminescence, light causes a substance to glow; fluorescence and phosphorescence are based on a substance’s ability to absorb light and emit light with a longer wavelength and lower energy. The main distinction is how long it takes them to complete the task. In fluorescence, the emission is basically immediate and thus generally only visible with the light source continuously found, so it disappears immediately without a light source, like a picture of a scene at a nightclub with teeth, eyes, and fabric glowing under a black light. While phosphorescent materials can store absorbed light energy for a period of time before releasing light after the light is turned off, the afterglow can last anywhere from a few seconds to hours depending on the phosphor’s material components, such as in an emergency exit sign. The glow in chemiluminescence is caused by a chemical reaction and requires activation, similar to a glow stick^6^.

Recently, luminous or high-definition phosphorescent hues in various shades have entered the fashion world for children’s and youth clothing to draw attention, innovation, and distinction, rather than as protective apparel. In addition to being utilized as decorative fabrics for interior design and textile tools for unique purposes. Researchers recently suggested that cotton cloth colored with fluorescent dyes could be used to detect and diagnose specific medical diseases, such as cancer cell activity, cellular toxicity indicators, and antimicrobial activity detection^7^.

Depending on the final applications in which high-visible fabrics are employed, each of them is subject to the technical specifications for the end application^8^. In particular, garment materials must meet comfort standards such as air permeability, water permeability, handling, and weight, in addition to functional qualities (durability of use) such as bursting or tensile strength, abrasion, and pilling. Aside from color fastness, there is also reflective visibility.

HVF is a modified cloth containing fluorescent elements that react to UV light. The fluorescent reaction is most evident in low-light environments where sight is limited. Although the fluorescent fabric dates back to 1930, it was identified in 1990 as the origin of hi-vis fabric. Also, various requirements of American and Indian.etc. of hi-vis fabric as protective apparel required to be flame resistant, and great attention was made to placing care labels on them to ensure increased efficacy and sustainability of use^9^. Nowadays, HVF or Hi-Vis fabric is considered as smart textiles^10^.

Hi-vis fabric is typically made from cotton 100%, silk, polyester, acrylic, polyamide, or cotton blend polyester, which is a frequent material in florescent fabric and is dyed with fluorescent dyes using various procedures depending on the nature of the materials^11,12^. More firms that create these fabrics use woven techniques with twill 2/2, 3/1, 4/1, and 3/4 structures, as well as knitting techniques with fleece structures, to produce fabrics ranging in weight from 180 to 300 g/m2 depending on the work environment and requirements^13^.

The purpose of this study is to use fluorescent dyes (disperse red and yellow) to dye two lightweight samples of knitted fabrics with a common structure of single jersey (plain) made of cotton/polyester and bamboo/polyester in a single dyeing step, in order to investigate the effect of fluorescent dyes on them and use this fabric in a high visible cloth for various functional garments. Previously, no research were conducted to dye bamboo using fluorescence dyes, as well as most high-visibility (hi-viz) textile items made from polyester or cotton/polyester with medium and heavy-weight woven fabrics.

This study introduces a novel approach to utilizing fluorescent dyes on bamboo-based fabrics, which, to date, have not been widely explored in the context of high-visibility applications. Unlike traditional high-visibility fabrics that primarily use polyester or cotton/polyester blends, this research examines the potential of bamboo/polyester blends dyed with fluorescent dyes. The focus on lightweight knitted fabrics rather than heavier woven fabrics expands the application possibilities for high-visibility clothing. Additionally, the research provides new insights into the effects of fluorescent dyes on both cotton and bamboo-based fabrics, contributing to the development of innovative, functional, and aesthetically diverse high-visibility garments.

## Materials and methods

### Materials

Two knitted samples with a single jersey (plain) structure were produced using a circular knitting machine (Mayer & Cie) with the following specifications: machine gauge [E] 20”, cylinder diameter [Inch] 26”, 42 feeders, arrangement yarn material feeding (one polyester yarn to one cotton or bamboo yarn), 1632 needles, and 85% efficiency rate. Cotton and bamboo with a yarn count of 30/1 Ne were combined with polyester 70 denier in a 50:50% ratio. Table 1 shows the sample code used in the results. Non-ionic detergent (Hostapal, acquired from Clariant). Sodium carbonate (NaCO3) and acetic acid (CH3COOH) were acquired from Lob Co. for chemicals in India.

**Fig. 1:**
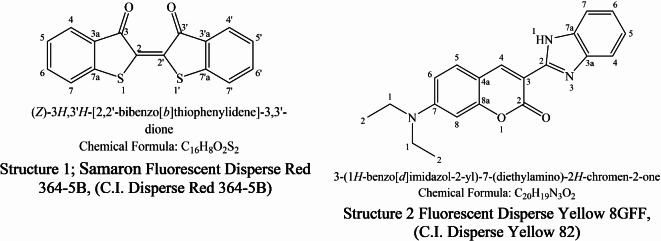
Chemical structure of florescence disperse dyes used.

Hang Zhou Fu Cai Co. Ltd., China, provided the dispersion dyes fluorescent pink (Samaron Fluorescent dispersion Red 364-5B), C.I. Disperse Red 364 (C16H8O2S2, λmax = 560), and fluorescent yellow 8GFF, C.I. Disperse Yellow 82 (C20H19O2N3, λmax = 450) (refer to Fig. 1).

**Table 1:** Description of sample code.

Modification	Sample code	Description of Sample
Blank	PB	Polyester/bamboo knitted fabric without dyeing
	PC	Polyester/cotton knitted fabric without dyeing
Disperse Red dye (Light Pink)	PB	Polyester/bamboo knitted fabric dyed with Disperse Red
	PC	Polyester/cotton knitted fabric dyed with Disperse Red
Disperse Yellow dye (Light Yellow)	PB	Polyester/bamboo knitted fabric dyed with Disperse Yellow
	PC	Polyester/cotton knitted fabric dyed with Disperse Yellow

### Methods

#### Fabric pretreatment

At a material-to-liquor ratio (MLR) of 1:40, the fabric was scoured in an aqueous solution comprising 2 g/L non-ionic detergent (Hostapal, Clariant) and 2 g/L sodium carbonate for 30 min at 50 °C. It was then allowed to dry overnight at room temperature.

#### Dyeing process

**Fig. 2:**
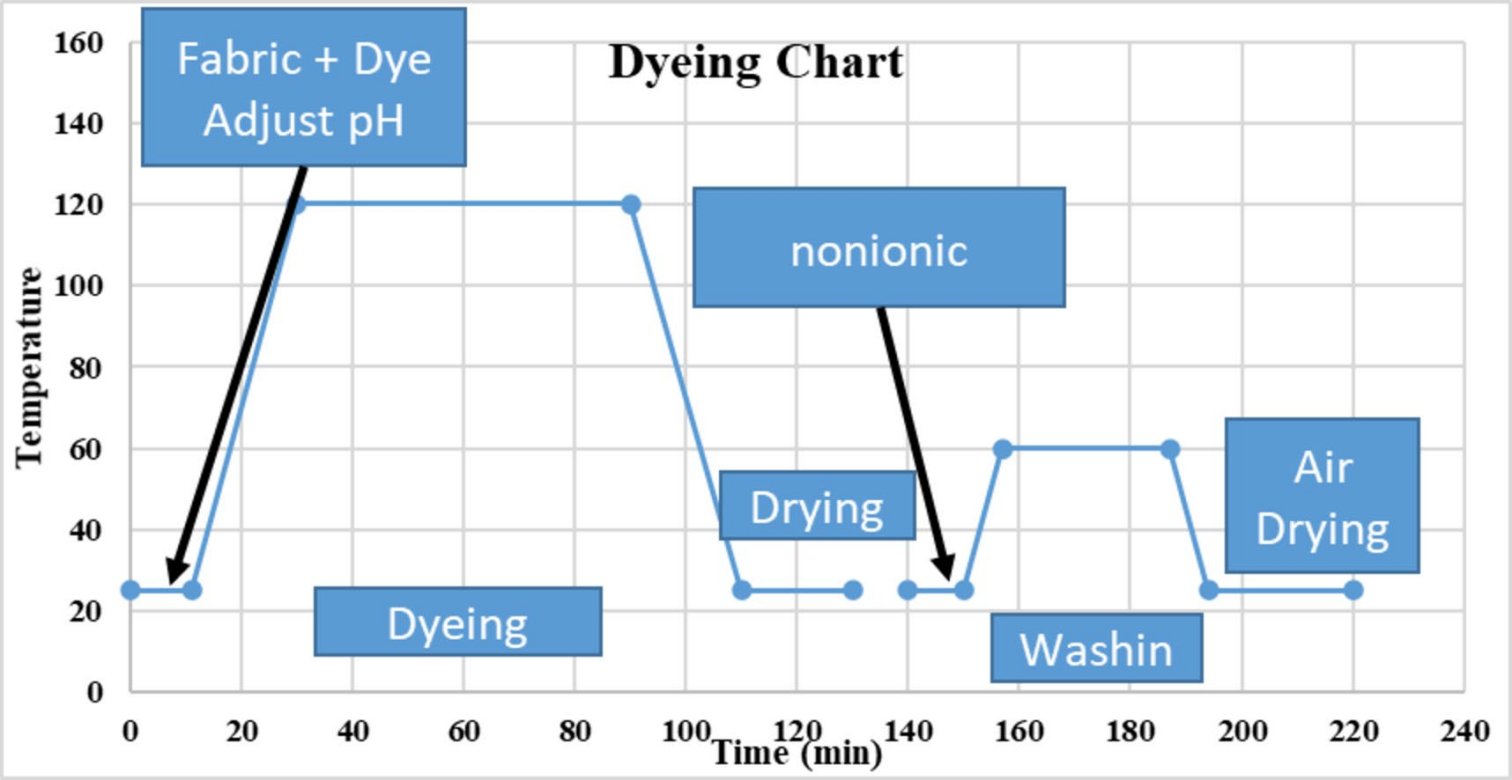
dyeing graph.

The fabric was colored in an IR dyeing machine (Infrared Color Dyeing Machine, Mumbai, India) with ML R 1:40 at 120 °C for one hour, utilizing varied dye concentrations of 0.5, 1, and 1.5%, as well as pH levels of 5, 7, and 9. Following the dyeing method, the colored fabric samples were immersed in a 60 °C solution containing 2% non-ionic detergent, then washed with water and allowed to dry naturally. Figure 2 depicted a dyeing graph from 14 to 16.

### Analyses and characterization

#### Color strength K/S

An analysis of the dyed samples was performed colorimetrically with a Hunter Lab Ultra Scan PRO spectrophotometer. The color strength/depth was represented by the K/S values, which were computed using the Kubelka-Munk Eq. (1)^14–20^, and ∆E values were calculated using Eq. (2).

1 $$\:\text{K}/\text{S}\:=\frac{{(1-\varvec{R})}^{2}}{2\varvec{R}}-\:\frac{{(1-{\varvec{R}}_{\varvec{o}})}^{2}}{2{\varvec{R}}_{\varvec{o}}}$$

Where; K is the absorption coefficient, S is the scattering coefficient, R is the decimal fraction of the reflectance of the dyed fabric, and R_o_ is the decimal fraction of the reflectance of the undyed fabric.

2 $$\Delta \:E_{ab}^* = \sqrt {\left( {L_2^* - L_1^*} \right)^2 + \left( {a_2^* - a_1^*} \right)^2 + \left( {b_2^* - b_1^*} \right)^2}$$

Delta E (the total color difference) is based on delta L*, delta a*, and delta b* color values, all of which provide a complete numerical descriptor of the color in a rectangular coordinate system. The meanings are as follows^21,22^:

- dL* represents a lightness difference between the sample and standard colors.
- da* represents the difference in redness or greyness between the sample and standard colors.
- db* denotes blueness-yellowness differences between the sample and standard colors.

#### Fastnesses properties

The color fastness of the dyed fabrics was assessed by the AATCC Test Method 16-2014 (color fastness to light), AATCC Test Method 61-2013 (color fastness to laundering/washing), and AATCC Test Method 8-2016 (color fastness to rubbing) and AATCC Test Method 15-2016 (color fastness to perspiration)^23–26^.

##### RUI measurement

The reflectance values of 10 randomly selected locations on the dyed sample were measured using a reflectance spectrophotometer in the visible spectrum region. The standard deviation (S) was determined using the equation below, while the mean of reflectance values for n wavelengths (R_m_) and the photopic relative luminous efficiency function (V) were calculated to determine the relative unlevelness index (RUI)^27^. The reflectance value of the measurement number i is represented by R_i_ for each wavelength.

$$\:\text{S}{\uplambda\:}\:=\sqrt{\frac{{\sum\:}_{i=1}^{n}{({R}_{i}-\:{R}_{m})}^{2}}{n-1}}\:\text{R}\text{U}\text{I}\:={\sum\:}_{\lambda\:=350}^{\lambda\:=700}(\frac{{S}_{\lambda\:}}{{R}_{m}}\times\:{V}_{\lambda\:})$$

Excellent levelness was defined as RUI < 0.2, while good levelness was defined as RUI < 0.49. Poor levelness is defined as RUI values between 0.5 and 1, while bad levelness is defined as RUI values greater than 1^28^.

#### Mechanical and physical properties

All samples before testing were kept relaxed and conditioned at a standard atmosphere for 24 h (temperature of 20 ± 2 °Cand relative humidity of 65 ± 2%), according to the standard test method for fabric ISO 139 ^29^. Consequently, the following mechanical and physical properties were tested to find out the effect of using both dispersed dyes on knitted blended samples as follow:

Mass per unit area (weight): Following the standard test method ASTM D 3776/D3776M − 09a (Reapproved 2017) utilizing an electronic digital balance^30^, Thickness test was estimated according to ASTM D1777-96:2017 utilizing Teclock thickness gauge^31^. The air permeability test was estimated according to ASTM D 737–2018 utilizing Toyoseiki (JIKA) instrument^32^, Water permeability test (Hydraulic pressure water) was estimated according to AATCC 127–2003, utilizing Toyoseiki (JIKA) instrument^33^, Bursting Strength: was estimated according to ASTM-D3786-01, utilizing M229 Auto burst tester SDL ATLAS tester^34^. Abrasion resistance: was estimated according to ASTM-D 3885-07, utilizing a Martindale tester^35^. Ultra Protective Factor was estimated according to AATCC 183:2010 ^36,37^ and Australian/New Zealand standard (AS/NZS 4366 − 1996)^38^. The ultraviolet transmission through the fabric was determined by a Cary Varian 300 UV-Vis spectrophotometer^39–44^. For each test, three replicates were being executed for blank and dyed samples, and the averages were calculated for all readings.

#### Fluorescent behavior evaluation

Visual and fluorescent visualization effects were observed by imaging the fabrics under D65 and UV light in the assessment light cabinet box (Verivide)^45^.

#### Evaluation and analytical study of the overall quality of the dyed fabrics

All results were collected, tabulated, and statistically analyzed using different tools. The paired t-test with a P-value ≤ 0.05 was performed to identify if the characteristics of manufactured knitted samples were affected significantly via the used treatment.

To select the best sample, the functional appropriateness of the produced fabric samples for research was evaluated. Thus, the radar chart areas multi-axis were computed to evaluate the quality factor of the research samples by transforming the average fabric property measurements into relative comparison values (without units), ranging from (0-100)^44^. The relative comparable value was determined using the following Eq. (3).

3 $$\:\text{Q}\text{F}\:=\frac{\mathbf{X}}{{\mathbf{X}}_{\mathbf{m}\mathbf{a}\mathbf{x}}}\:\times\:100\:\text{o}\text{r}\:\text{Q}\text{F}\:=\frac{{\varvec{X}}_{\varvec{m}\varvec{i}\varvec{n}}}{\varvec{X}}\:\times\:100$$

Where; X (read each sample separately), X_Max_ (highest reading), and X_Min_ (lowest reading).

## Results and discussion

Both produced knitted fabrics (polyester/cotton and polyester/bamboo) were dyed using two fluorescence dyes. Various chemical, mechanical, and physical properties of the fabrics were carried out.

### Optimalisation of the dyeing properties

#### Effect of pH

**Fig. 3:**
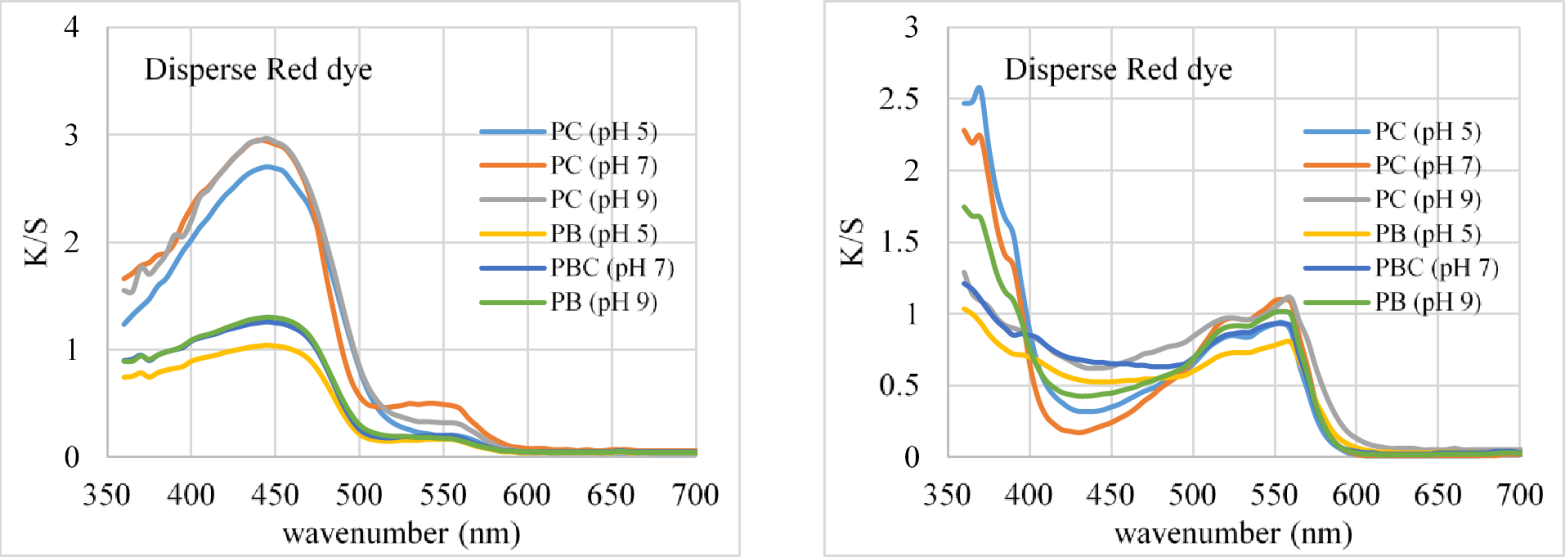
Color strength of different fabrics dyed using different dyes at different pH.

One of the most critical parameters influencing dye absorption into fabrics was the pH of the dye bath. Table 2; Fig. 3 show the color strength (K/S) and levelness value (RUI) of two colored polyester blended textiles (polyester/cotton and polyester/bamboo) with dispersed dyes at different pH levels. The color strength of both polyester blended materials colored with each disperse dye increases with increasing pH in the dying water. The ideal pH for both dispersed dyes and fluorescence dyes in blended fabrics is 7. As indicated by the K/S and (E values. Furthermore, the L*, a*, and b* values vary slightly as the pH varies, particularly with polyester/bamboo samples. Furthermore, the RUI value demonstrated exceptional and good dye levelness with both dyes on both studied materials colored at varied pH levels. Furthermore, polyester/bamboo fabric exhibits greater dye levelness than polyester/cotton fabric.

**Table 2:** Effect of pH on the color strength.

Fabrics	pH	Disperse Yellow dye							Disperse Red dye						
		K/S	L*	a*	b*	∆E	RUI		K/S	L*	a*	b*	∆E	RUI	
							value	rank						value	rank
Polyester /cotton	5	2.73	70.79	11.96	45.08	53.84	0.186	E	0.87	69.36	30.43	11.74	35.58	0.307	G
	7	2.91	72.98	12.33	46.47	55.51	0.307	G	1.08	71.50	31.37	12.10	36.68	0.307	G
	9	2.93	73.34	12.39	46.70	55.79	0.262	G	1.11	71.86	31.53	12.16	36.86	0.171	E
Polyester /bamboo	5	1.05	77.87	13.67	37.28	44.43	0.171	E	0.82	75.68	24.61	6.36	26.52	0.222	G
	7	1.24	80.28	13.78	38.43	45.80	0.171	E	0.91	78.02	25.37	6.56	27.34	0.262	G
	9	1.26	80.68	13.80	38.62	46.03	0.174	E	0.98	78.41	25.50	6.59	27.48	0.289	G

Different pH used at 120 °C for 1 h, L: R 1:40.

L*:(lightness/darkens), a*:(redness/greenness), b*:(yellowness/blueness), **∆E**: (overall color difference values in Hunter L, a, b coordinates).

RUI values range can be categorized into If RUI < 0.2 that considered as excellent levelness. (E). If 0.2 < RUI < 0.49 that is considered as good levelness. (G), If RUI is between 0.5 and 1 that means poor levelness. (P), If RUI values are greater than 1 that indicate bad levelness. (B).

#### Effect of dye concentration

More discussion on how the color strengths (K/S) of all materials grow with dye concentration. These are expected results since the dye had more particles to enter and distribute throughout the fibers, allowing it to attain its full dye absorption capacity at the dynamic dyeing equilibrium state.

**Fig. 4:**
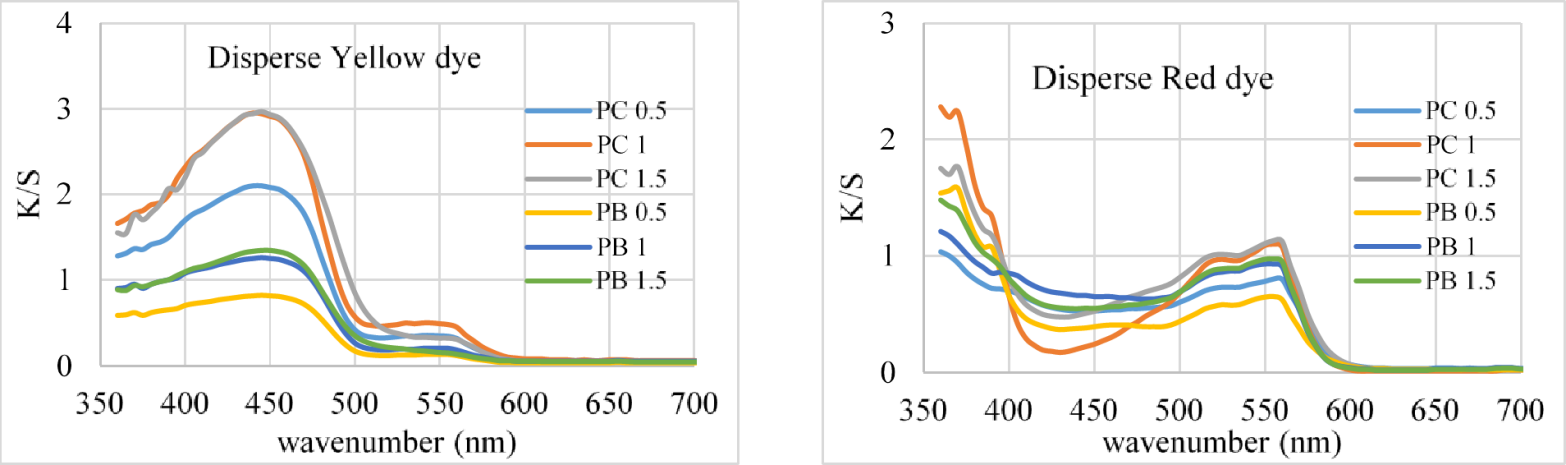
Color strength of different fabrics dyed using dyes (kind and concentration).

Table 3; Fig. 4 show the color strength (K/S) values for both dyed fabrics using fluorescent dyes at varying dye concentrations (0.5, 1, and 1.5%). Dyeing using Disperse Yellow dye produces a stronger color than Disperse Red dye, and raising the dye concentrations increases the K/S of both mixed polyester materials. The K/S and ∆E values show that both fabrics colored with fluorescent dyes have excellent K/S and ∆E values at 1% dye concentration. Increasing the dye concentration slightly increases both K/S and ∆E values.

Furthermore, the RUI value demonstrated outstanding and good dye levelness using both dyes on both studied materials dyed at varying dye concentrations. Furthermore, polyester/bamboo fabric exhibits greater dye levelness than polyester/cotton fabric.

The relationship between dye concentration and color strength (K/S) is a critical factor in achieving the desired dyeing results for fabrics. In this study, the increase in K/S values with higher dye concentrations is consistent with the fundamental principles of dyeing: as more dye is available in the dye bath, more dye molecules can penetrate the fibers, fill available sites, and interact with the fabric matrix. This results in deeper and more vibrant coloration, as seen across the tested samples.

Comparison of Disperse Yellow and Red Dyes: a) Dye Absorption and Fiber Interaction: Disperse Yellow dye exhibited a higher color strength compared to Disperse Red dye across all concentrations. This could be attributed to the specific molecular structure of Disperse Yellow, allowing for better penetration and binding within the fiber matrix, possibly influenced by its affinity for polyester blends in the context of hydrophobic interactions and surface adsorption, B) Saturation and Equilibrium: At lower concentrations (0.5%), the differences in K/S values between yellow and red dyes were more pronounced. As the concentration increased to 1%, both dyes achieved significant saturation levels, reaching dynamic dyeing equilibrium. At this point, the fabrics displayed optimal color strength and uniformity. Beyond a 1% concentration, the incremental gains in color strength and ∆E values were minimal. This suggests that the dyes had saturated most available binding sites in the fibers, and additional dye could not significantly enhance the color intensity due to limited unoccupied sites and the potential aggregation of excess dye, C) Implications for Practical Applications: These findings highlight the efficiency of achieving desired color strength with lower dye concentrations, which can be cost-effective and reduce environmental impact from excess dye runoff. The study suggests an optimal concentration of 1% as a balance between achieving strong color intensity and maintaining economic and environmental standards in the dyeing process, and D) Overall Effects and Observations: The color uniformity observed at the optimal dye concentration also suggests good dye distribution within the fabric, crucial for high-visibility applications where consistent luminescence is required.

These insights provide valuable information for manufacturers looking to optimize dyeing processes, select appropriate dyes, and achieve consistent, high-quality results for high-visibility apparel and other applications requiring vibrant and durable colors.

**Table 3:** Effect of dye concentration on the color strength.

Fabrics	Dye conc.	Disperse Yellow dye							Disperse Red dye						
		K/S	L*	a*	b*	∆E	RUI		K/S	L*	a*	b*	∆E	RUI	
							value	rank						value	rank
Polyester /cotton	0.5	2.03	69.33	11.71	44.15	52.73	0.266	G	0.80	67.93	29.80	11.50	34.85	0.222	G
	1	2.91	72.98	12.33	46.47	55.51	0.307	G	1.08	71.50	31.37	12.10	36.68	0.307	G
	1.5	2.95	73.34	12.39	46.70	55.79	0.262	G	1.13	71.86	31.53	12.16	36.86	0.270	G
Polyester /bamboo	0.5	0.81	76.27	3.59	36.51	43.51	0.171	E	0.62	74.12	24.10	6.23	25.97	0.222	G
	1	1.24	80.28	3.78	38.43	45.80	0.171	E	0.91	78.02	25.37	6.56	27.34	0.262	G
	1.5	1.27	80.68	3.80	38.62	46.03	0.177	E	0.95	78.41	25.50	6.59	27.48	0.277	G

Different dye concentrations used at pH (7), 120 °C for 1 h, L:R 1:40.

L*:(lightness/darkens), a*:(redness/greenness), b*:(yellowness/blueness), **∆E**: (overall color difference values in Hunter L, a, b coordinates).

RUI values range can be categorized into If RUI < 0.2 that considered as excellent levelness. (E). If 0.2 < RUI < 0.49 that is considered as good levelness. (G), If RUI is between 0.5 and 1 that means poor levelness. (P), If RUI values are greater than 1 that indicate bad levelness. (B).

Finally, Tables 2 and 3 summarize the impacts of pH variations and varied dye concentrations, demonstrating that both polyester blended materials were given a homogeneous treatment, resulting in K/S values for the colored fabrics. The effect of dyeing polyester blended materials with both dispersion fluorescent dyes on color strength was examined^46–48^.

##### Color fastness measurements

The color persistence of polyester blended fabrics treated with both dispersed fluorescent dyes was evaluated based on washing and light fastness. Table 4 shows the color fastness of dyed polyester blended samples. The range for both polyester blend textiles was 4–6. Furthermore, it is clear that the light fastness varies from fair to exceptional in all cases.

**Table 4:** Fastness properties of the dyed polyester blended samples.

Modification	Sample code	Washing Fastness	Light fastness
Disperse Red dye (Light Pink effect)	PB	3	4–5
	PC	5	6–7
Disperse Yellow dye (Light Yellow effect)	PB	3	4–5
	PC	4	6–7

##### Ultraviolet protection (UPF)

Table 5 shows the UPF values of blank and colored polyester blended samples treated with two types of dispersion dyes. The results show that bamboo/polyester samples had a greater UPF value than cotton/polyester samples. This is consistent with earlier research and verifies bamboo’s impact on UV wave protection^44,49,50^.

Furthermore, all dyed samples exhibit greater UPF values than blank (undyed) samples, confirming that disperse dye shields from UV rays; however, this protection is dependent on the wavelength of the color; as the color becomes darker, the fabric delivers a higher UPF value. This idea is consistent with the results, as both blended samples with disperse red dye have greater UPF values than samples with scatter yellow dye. This impact could be due to the way dye molecules are designed to absorb UV radiation, which improves the ability of colored materials to block UV radiation, offering protection and giving human skin a new ability to shield against UV radiation.

**Table 5:** UPF values of blank and dyed polyester blended fabrics with both disperse dyes before and after washing.

Modification	Sample code	UPF	
		Before washing	After washing
Blank	PB	11.19 ± 0.03	11.21 ± 0.03
	PC	7.19 ± 0.03	7.2 ± 0.03
Disperse Red dye (Light Pink effect)	PB	13.26 ± 0.47	15.13 ± 0.93
	PC	9.72 ± 0.34	11.09 ± 0.68
Disperse Yellow dye (Light Yellow effect)	PB	11.3 ± 0.4	12.89 ± 0.8
	PC	7.26 ± 0.26	8.28 ± 0.51

### Characterization of treated fabrics

#### Structural properties

Table 6 shows the specs for both blank and dyed samples. The data show that dyed fabrics tend to encourage more shrinkage in knitted fabrics with increased wales and courses per centimeter, which has an impact on loop length and tightness factor 51. Furthermore, stitch density results show that dispersed yellow dye is more effective on manufactured knitted fabrics than dispersed red dye.

Furthermore, the data indicate that blending bamboo/polyester has a greater tendency to contract its loop length on knitted fabric (in a relaxed condition) than blending cotton/polyester, whether on a blank or after dyeing. The cause can be traced to the material’s specific density, which is around 1.54 g/cm^3^ for bamboo and 0.8 g/cm^3^ for cotton^51,52^. The higher the specific density of the material, the more moisture is regained, resulting in lower fiber length (cellulose) of the formed yarn and therefore reducing overall yarn length, which influences loop length and, consequently, tightness factor.

**Table 6:** Specifications of blank & dyed knitted samples.

Modification	Sample code	Wales/cm	Courses/cm	Loop Length [mm]	Stitch Density [stitches/cm^2^]	Tightness Factor [√tex/mm]
Blank	PB	12	18	2.8	216	1.58
	PC	12	17	2.9	204	1.52
Disperse Red dye (Light pink effect)	PB	14	20	2.3	280	1.92
	PC	13	19	2.6	247	1.70
Disperse Yellow dye (Light yellow effect)	PB	14	21	2.2	294	2.01
	PC	13	20	2.5	260	1.77

#### Physio-mechanical and comfortable properties

Table 7 compares the qualities of blank and dyed polyester blended samples. The findings describe the variable behavior of samples before and after dyeing, in which structural properties (weight and thickness) were increased, comfort properties (air and water permeability) were slightly affected, and mechanical properties (bursting strength and abrasion resistance) were decreased. Furthermore, the results show that dyeing has a greater impact on the characteristics of bamboo/polyester knitted samples than on cotton/polyester knitted samples. The interpretation is based on a change in sample specification (as given in Table 6), which has an impact on stitch densities per area, yarn intermeshing, and pore size.

**Table 7:** Characteristics of blank & dyed knitted samples.

Modification	Sample code	Weight [g/m^2^]	Thickness [mm]	Air permeability [cm^3^/cm^2^.s]	Water permeability [L/ cm^3^.s]	Bursting strength [Kpa]	Abrasion (weight loss) [%]
Blank	PB	131.90	0.53	276.37	1.02	506.67	0.006
	PC	82.17	0.50	325.06	1.03	503.33	0.005
Disperse Red dye (Light Pink effect)	PB	169.30	0.68	107.72	0.94	498.00	0.008
	PC	105.33	0.68	167.80	0.96	452.50	0.007
Disperse Yellow dye (Light Yellow effect)	PB	169.50	0.69	95.60	0.93	501.33	0.014
	PC	105.87	0.70	96.95	0.97	461.50	0.008

##### Mass per unit area (weight)

**Fig. 5:**
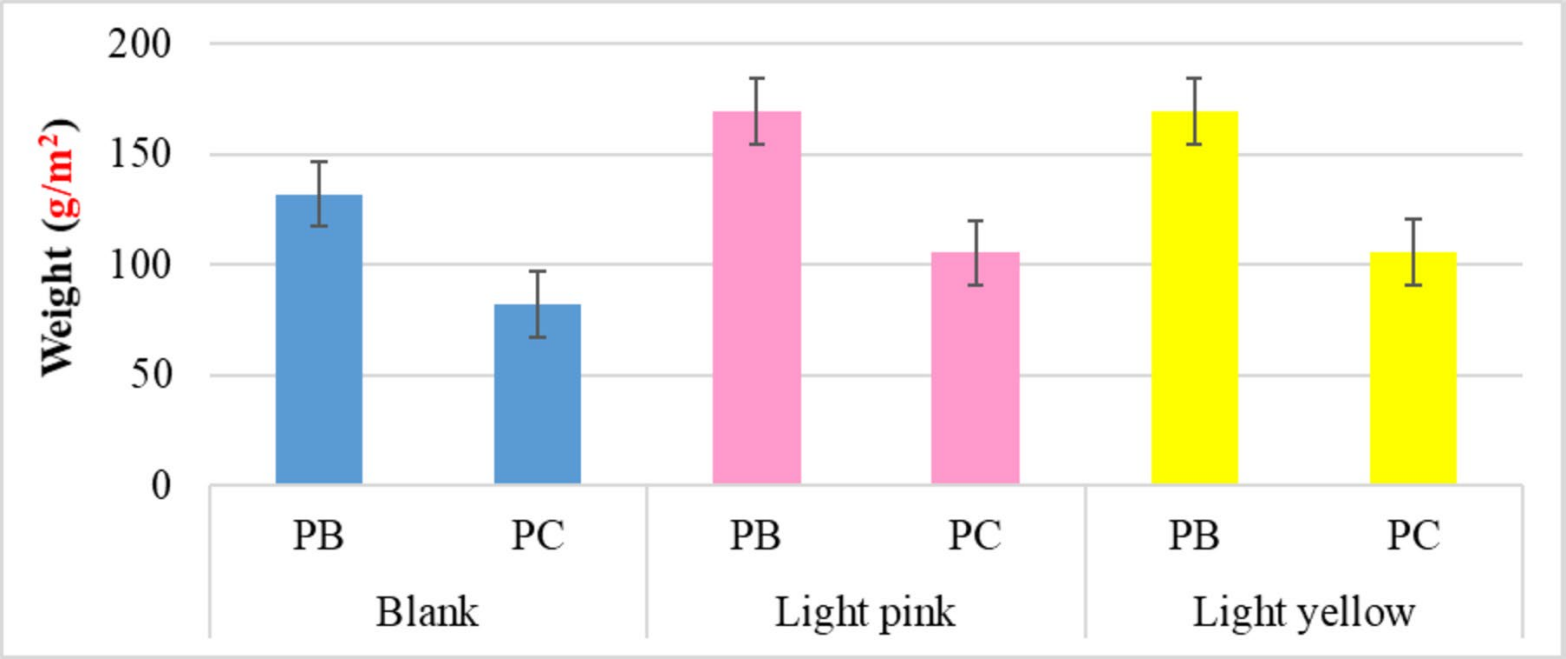
Weight values of the blank and dyed knitted samples.

Figure 5 shows how the various dyeing techniques influenced the manufactured samples. For example, using the dispersed red dye increased the mass per unit area (g/m2) by approximately 28.35% and 28.18% for bamboo/polyester and cotton/polyester, respectively. On the other hand, the growing rate with the distributed yellow dye was roughly 28.50 and 28.84% for blended polyester knitted fabrics with bamboo or cotton, respectively. Furthermore, the findings reveal that each of the fluorescent dyeing procedures has a rapprochement effect on the weight property of the same blended materials, indicating the closeness of the shrinkage effect.

##### Thickness (fabric height)

**Fig. 6:**
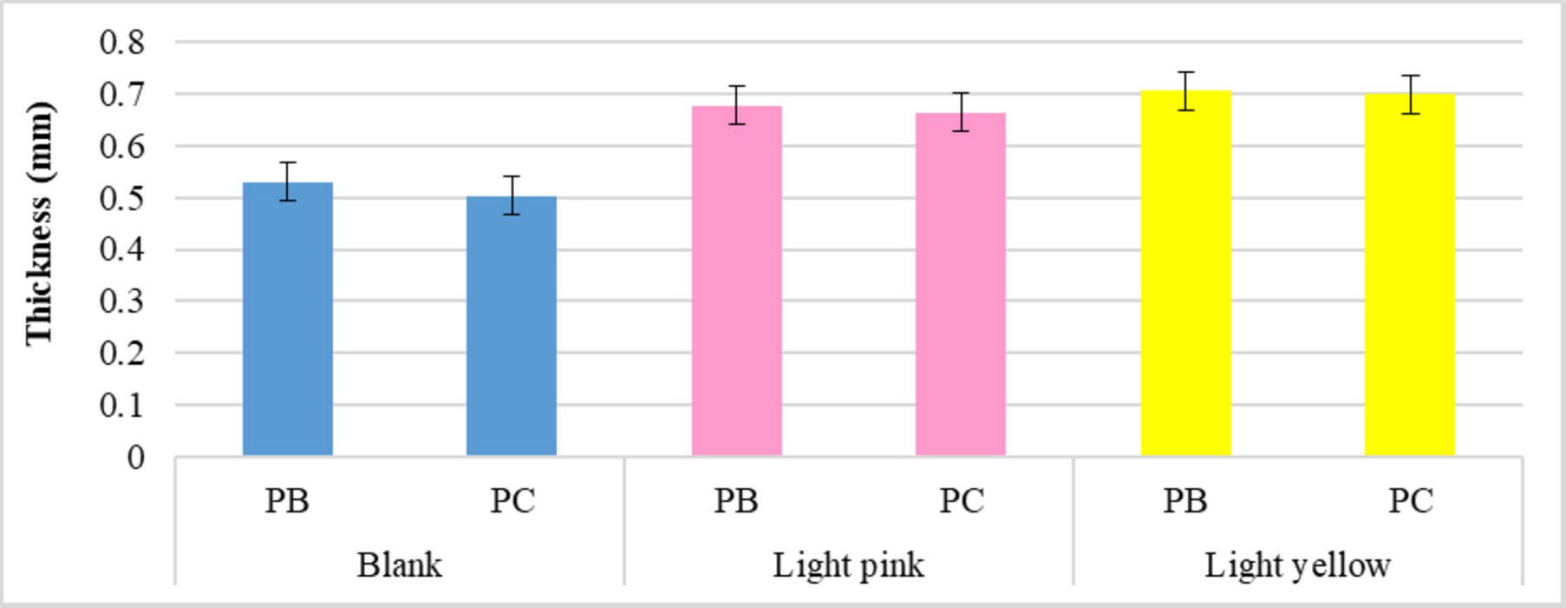
Thickness values of the blank and dyed knitted samples.

Figure 6 shows how dyeing procedures affect the thickness of knitted samples. The findings indicated an increase in sample thickness (mm) following dyeing processes, as well as the superior capability of disperse yellow dye on yarn swelling enhancement due to higher saturation compared to disperse red dyeing processes, which is consistent with the weight evaluation results.

##### Air permeability

**Fig. 7:**
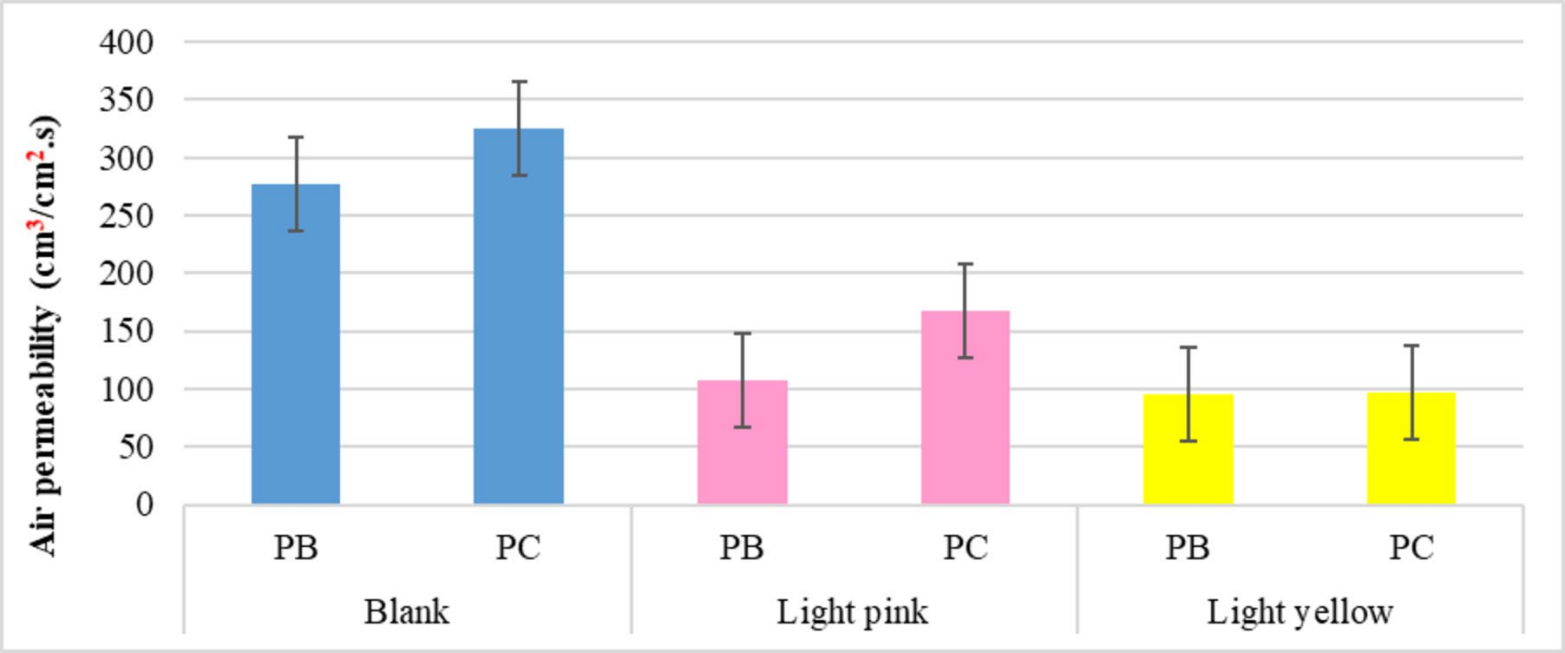
Air permeability rats of the blank and dyed knitted samples.

Based on the data values shown in Fig. 7, it is clear that airflow through produced samples was significantly impacted following dyeing procedures, with air permeability decreasing either with dispersed yellow or dispersed red dyes. In the same context, the data highlight the differences in the effect of the disperse red dye on the generated knitted samples, as opposed to the disperse yellow dye, which has a nearly identical effect. Indicating the variation in crosslinking for the used dispersed dyes with the blended knitted samples, wherein the red dye forms a stronger link with the bamboo/polyester sample than with the blended cotton/polyester sample, while the dispersed yellow dye performs roughly the same linking (interaction) with both.

Overall, the findings denote that the samples produced with blending polyester/bamboo yarns have been more influenced (reduced in air permeability) than those produced with blending polyester/cotton yarns, which could be traced to minimizing pores size at the fabric surface as a result of the fibers swelling and deposition of dye particles on the fabric surface during the dyeing process, especially with polyester material and bamboo (viscos bamboo) that considered a regenerate Furthermore, it was discovered that disperse yellow dye decreased the air permeability rate compared to disperse red dye for each sample. This could be attributed to the lower density of disperse yellow dye (1.2 g/cm^3^) compared to disperse red dye (1.54 g/cm^3^), which allows the yellow dye to swell within fibers and disperse widely on the fabric surface, reducing the area of fabric surface pores.

##### Water permeability

**Fig. 8:**
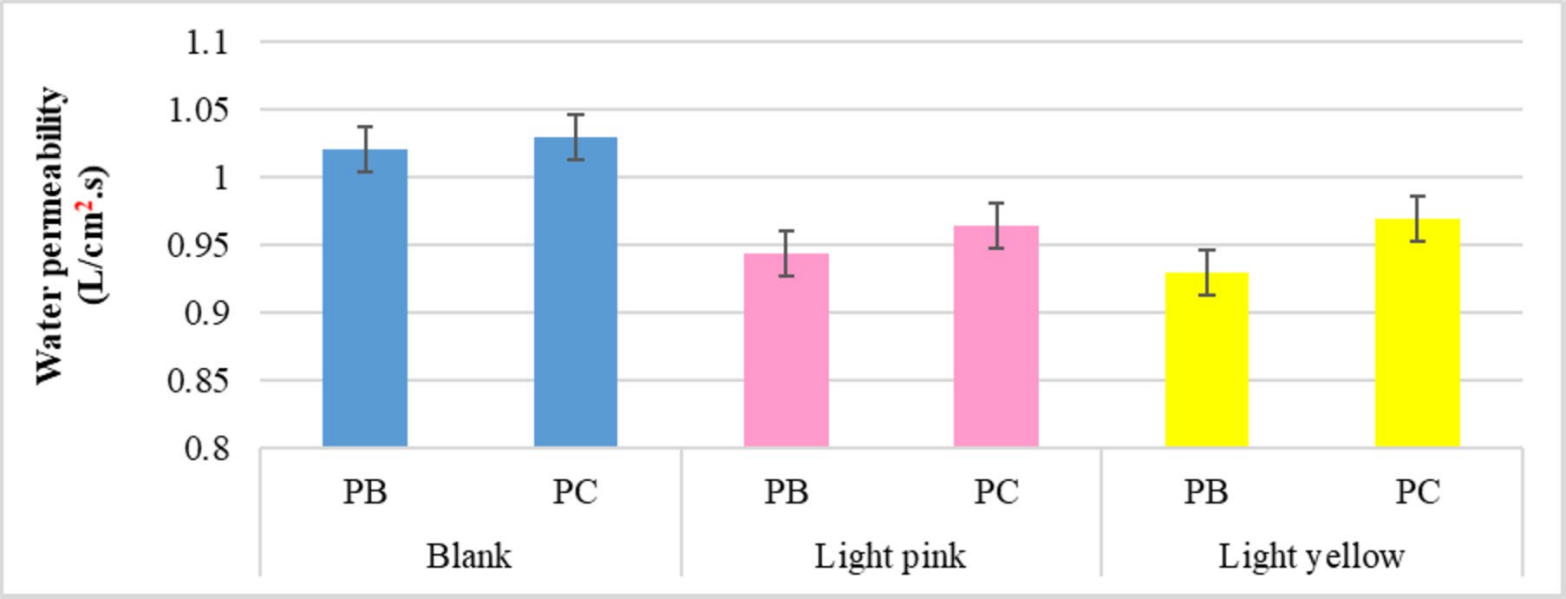
Water permeability rates of the blank and dyed knitted samples.

Figure 8 shows how the blank and dyed polyester mix samples performed in terms of water permeability. The findings state that the impact of dye properties during dyeing processes on the produced samples is that the water permeability of the dyed polyester blended samples decreases as a result of the dye molecules depositing on the fabric surface of the cellulosic material and some of them absorbing within the fibers occurred primarily inside the polyester fabric and partially in cellulosic materials. Furthermore, the dyed fabric produces similar results regardless of the type of dye used. In the same context, the findings demonstrate the effect of the nature of the blended materials, as polyester/bamboo knitted samples, whether blank or dyed, can provide more water retention than polyester/cotton, which can return to specific density (which is higher in bamboo) and low porosity (due to the increase in loop length as referred to in Table 7).

##### Bursting strength resistance

**Fig. 9:**
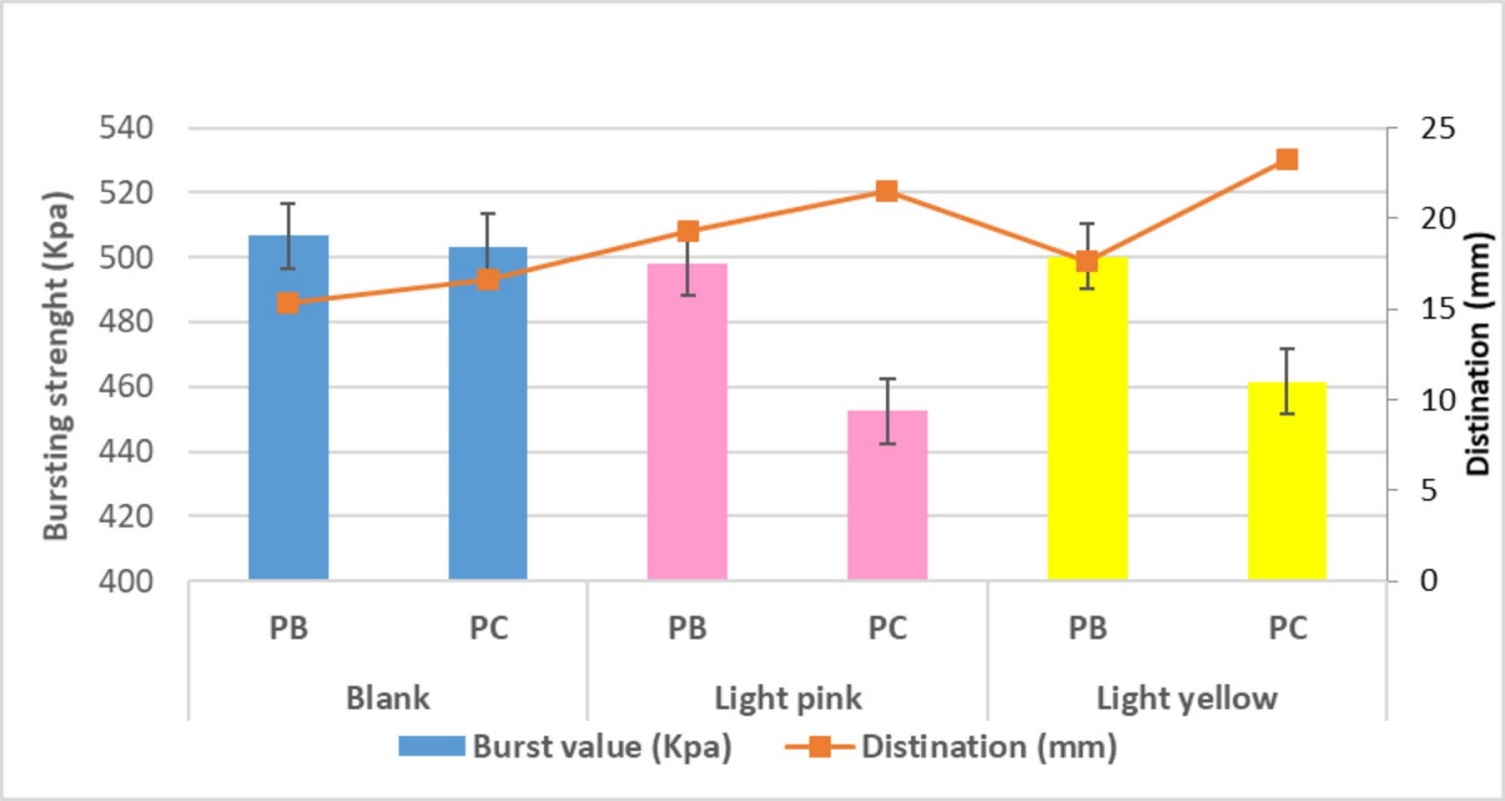
Bursting strength and Destination values of the blank and dyed knitted samples.

Depending on the computed results of the bursting strength test, as shown in Fig. 9. Dyeing has a significant impact on made knitted samples. When the resistance of samples was reduced while the destination (extension) was adjusted for different blended materials, it was discovered that there is an indirect relationship between bursting resistance and destination values for blank and colored samples. Furthermore, the data show that the blending of polyester/cotton samples was significantly changed (whether to prevent bursting or increase extension) when compared to the blending of polyester/bamboo samples using various dyes. Indicating that the cellulose link chain arrangement of cotton was significantly affected than bamboo for both dyes used, declaring the strong links that formed among the fluorescent disperse dyes man-made materials, so the resistance polyester/bamboo samples of burst strength higher than polyester/cotton.

##### Abrasion resistance

**Fig. 10:**
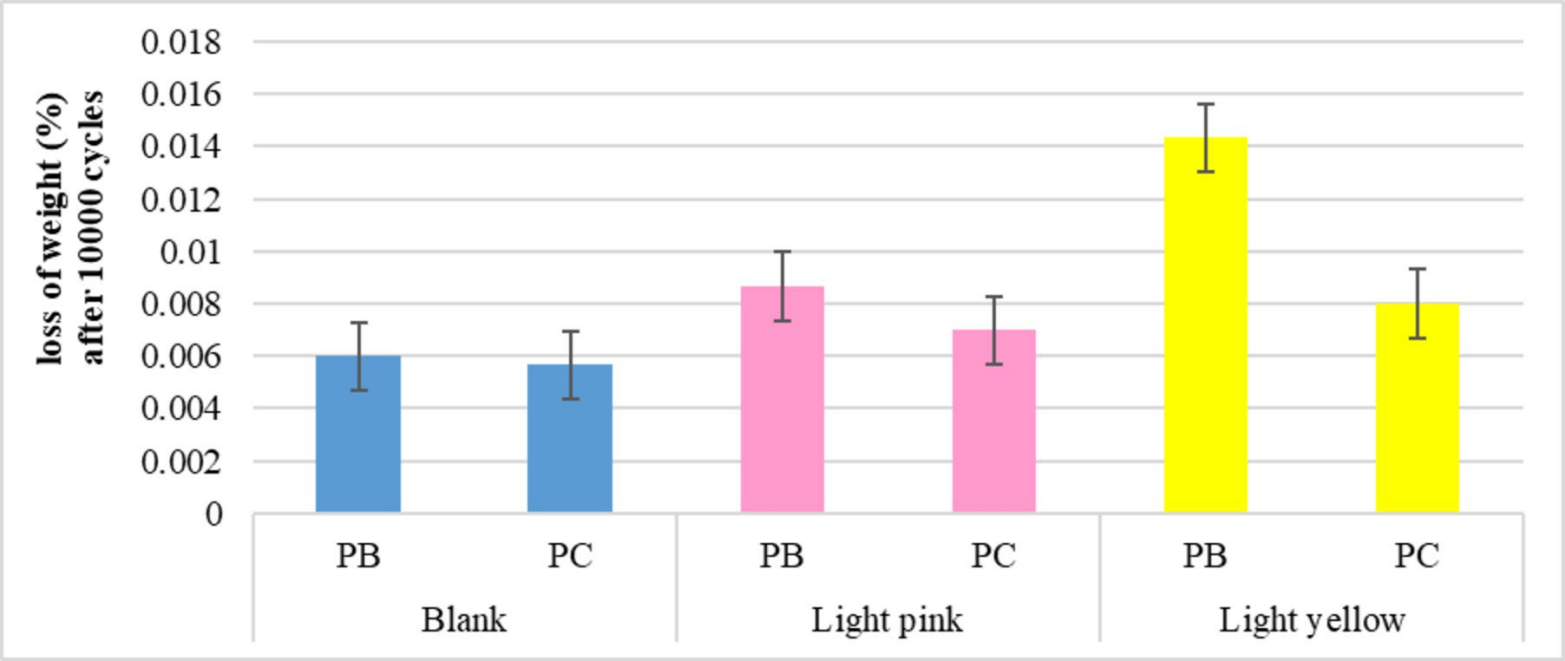
Abrasion resistance characteristic of the blank and dyed knitted samples.

The dyed knitted samples demonstrated inferior abrasion resistance (with higher weight loss%) than the blank samples. The rationale could be connected to an increase in stitch density per area exposed to friction as the colored samples contract in a relaxed state. Furthermore, the results showed that the blended knitted samples dyed with dispersion yellow dyeing had a stronger effect on reducing abrasion resistance than the blended knitted samples dyed with disperse red dye. This effect could be attributed to the yarns inflating as a result of the higher saturation quantity, particularly in colored polyester and bamboo samples. Figure 10 depicts the abrasion resistance characteristic of the blank and colored knitted samples, as shown by weight loss after 10,000 cycles.

### Evaluation of all characteristics measured

#### Significant effects of dyeing processes

To determine whether the utilized dye had a significant or non-significant effect on the resulting blended knitted samples, p-values from paired t-tests were calculated, as shown in Table 8. Overall, the data indicated that utilizing different fluorescent colors had a substantial impact on the various features of blended knitted fabrics, with the exception of bursting strength resistance for mixed polyester/bamboo. Furthermore, in terms of abrasion surface resistance, the findings show that using only the dispersed yellow dye had a significant effect on both blended polyester knitted fabrics with bamboo or cotton, which could be traced back to the efficacy of the dispersed yellow dyeing in the blended knitted samples, providing a high thickness due to the yarns’ swelling and, as a result, a more frictional surface.

**Table 8:** P-value of paired t-test for dyed samples.

Characteristics	Disperse Red Dye		Disperse Yellow Dye	
	Polyester/Bamboo	Polyester/Cotton	Polyester/Bamboo	Polyester/Cotton
Bursting strength	0.323247 ^NS^	0.004455*	0.775521 ^NS^	0.010235*
Thickness	0.003292*	0.028746*	0.00301*	0.004118*
Weight	0.000144*	0.000077*	0.000145*	0.000051*
Air permeability	0.000097*	0.000463*	0.000013*	0.000040*
Water permeability	0.006312*	0.005088*	0.004677*	0.00593*
Abrasion resistance	0.119958 ^NS^	0.207882 ^NS^	0.019804*	0.047191*

^(NS)^ Non-Significant Effect (p-value more than 0.05).

^(*)^ Significant Effect (p-value equal to or less than 0.05).

#### Radar chart area

**Fig. 11:**
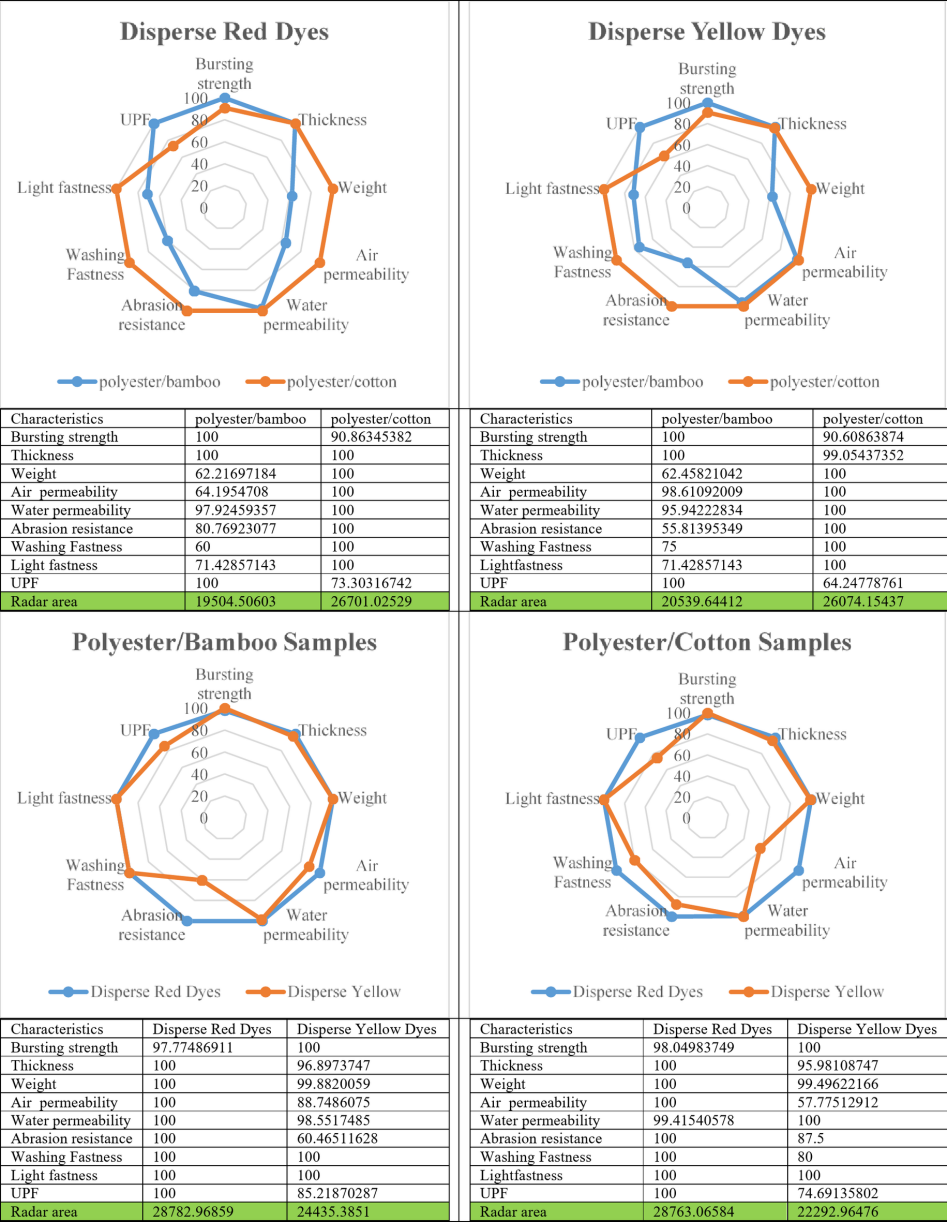
Radar chat area of produced samples.

To detect and arrange the produced blended knitted samples based on desirable performance after dying, the radar chart area was graphed and calculated, as shown in Fig. 11. The results show that disperse dyeing is more effective in improving the overall performance of knitted polyester samples blended with cotton than bamboo. Meanwhile, the findings show that the dyeing processes have a different impact on the performance of the produced blended knitted samples, with the dispersed red dye outperforming the dispersed yellow dye, referring to the differences in nature characteristics between cotton and bamboo, despite the fact that they are both composed of cellulosic materials.

### Evaluation of fluorescence color effect

**Fig. 12:**
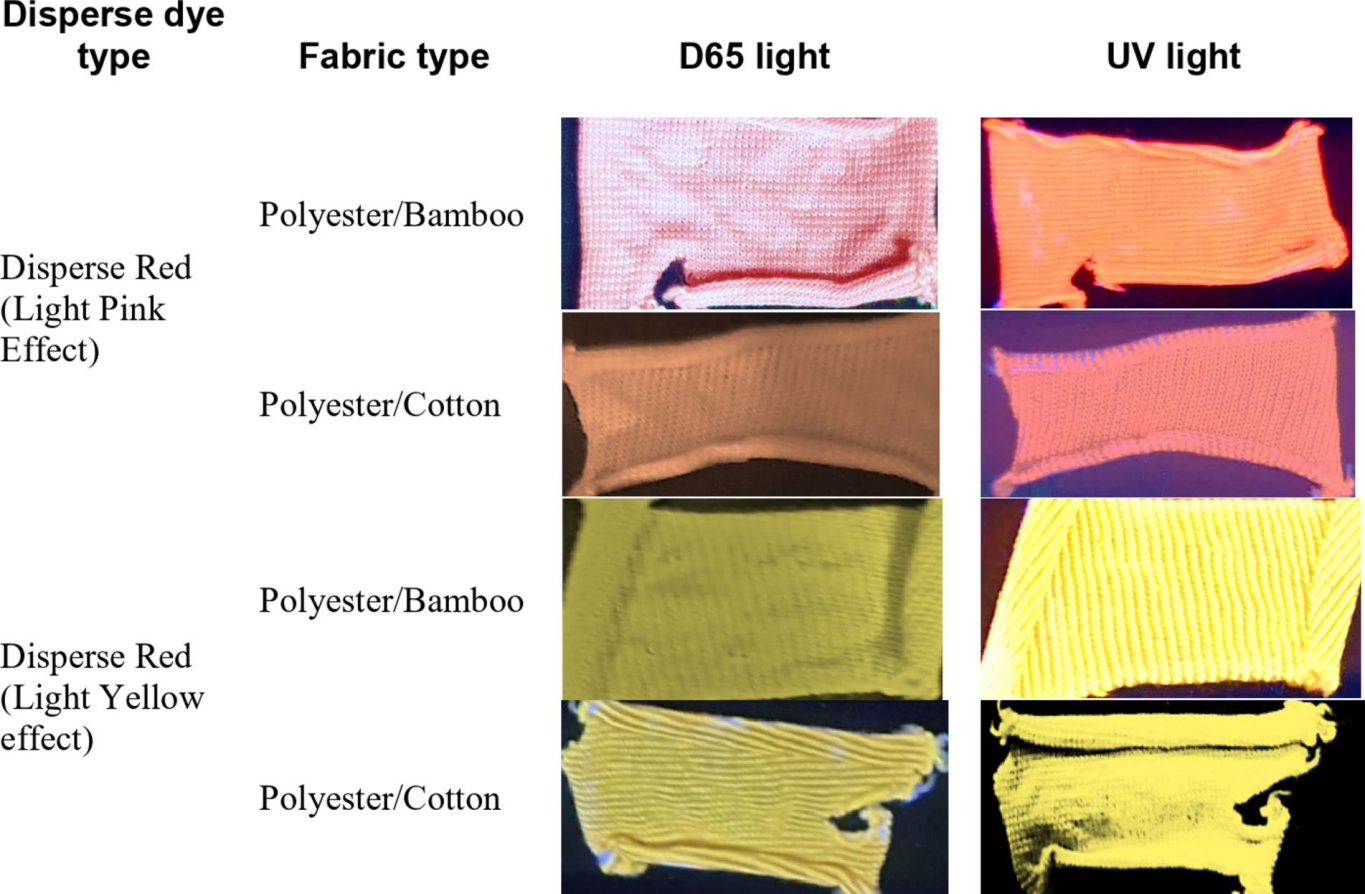
Optical and fluorescence images of both dyed fabrics before and after exposure to fluorescent light.

The excitation effect of blended polyester fabrics with bamboo or cotton dyed with dispersion fluorescent dyes has been studied for usage in the workplace or in traffic/racing applications. The polyester blended materials were dyed with two dispersed fluorescent dyes. Figure 12 displays a fluorescence optical picture before and after UV light treatment. Blended polyester fabric with bamboo allows for improved visibility than blended polyester fabric with cotton. Furthermore, dyed samples with disperse red provide a stronger fluorescence effect than dyed samples with disperse yellow.

## Conclusion

Functional dyes now have numerous applications in the textile industry, and fluorescent dyes are among those that have taken over. Two types of luminous dyes, Disperse Yellow 82 and Disperse Red 5B, were employed in this study to dye knitted fabrics with a single jersey (simple) structure made of polyester/cotton and polyester/bamboo. The findings for various functional properties were summarized as follows: (A) The color fastness against washing and light of dyed cloth samples with both distributed fluorescent dyes ranged from 4 to 6, (B) Polyester/bamboo samples had greater UPF values than cotton/polyester samples. Furthermore, all colored samples have greater UPF ratings than blanks. Additionally, blended samples with scatter red dye have a higher UPF rating than those with disperse yellow dye, (C) Dyeing procedures had a substantial impact on the mechanical and physical qualities of blended knitted fabrics, with the exception of polyester/bamboo bursting strength resistance, (D) The dispersion yellow coloring techniques had a substantial effect on the abrasion resistance of both blended polyester knitted fabrics with bamboo or cotton, (E) The radar area shows that scattered dyeing is more effective at improving the overall performance of knitted polyester/cotton than polyester/bamboo. Similarly, the results show that distributed red dyeing has a significantly greater impact on the generated knitted samples than dispersed yellow dyeing, and (F) After UV exposure, the polyester/bamboo samples are more visible than the polyester/cotton samples. Furthermore, dyed samples with disperse red provide a stronger fluorescence effect than dyed samples with disperse yellow.

## Data Availability

The datasets generated and/or analysed during the current study are available in the manuscript.
